# Supplementary material for: Novel Driver Strength Index highlights important cancer genes in TCGA PanCanAtlas patients
Source: PeerJ. 2022 Aug 11;10:e13860. doi: 10.7717/peerj.13860 (PMC9375969; doi:10.7717/peerj.13860)
Supplement: Supplemental Information 2 [file peerj-10-13860-s002.zip › COHORTS/genes plots/cohorts/2021_8_16_14_9_distribution_cohorts_LUAD.pdf]

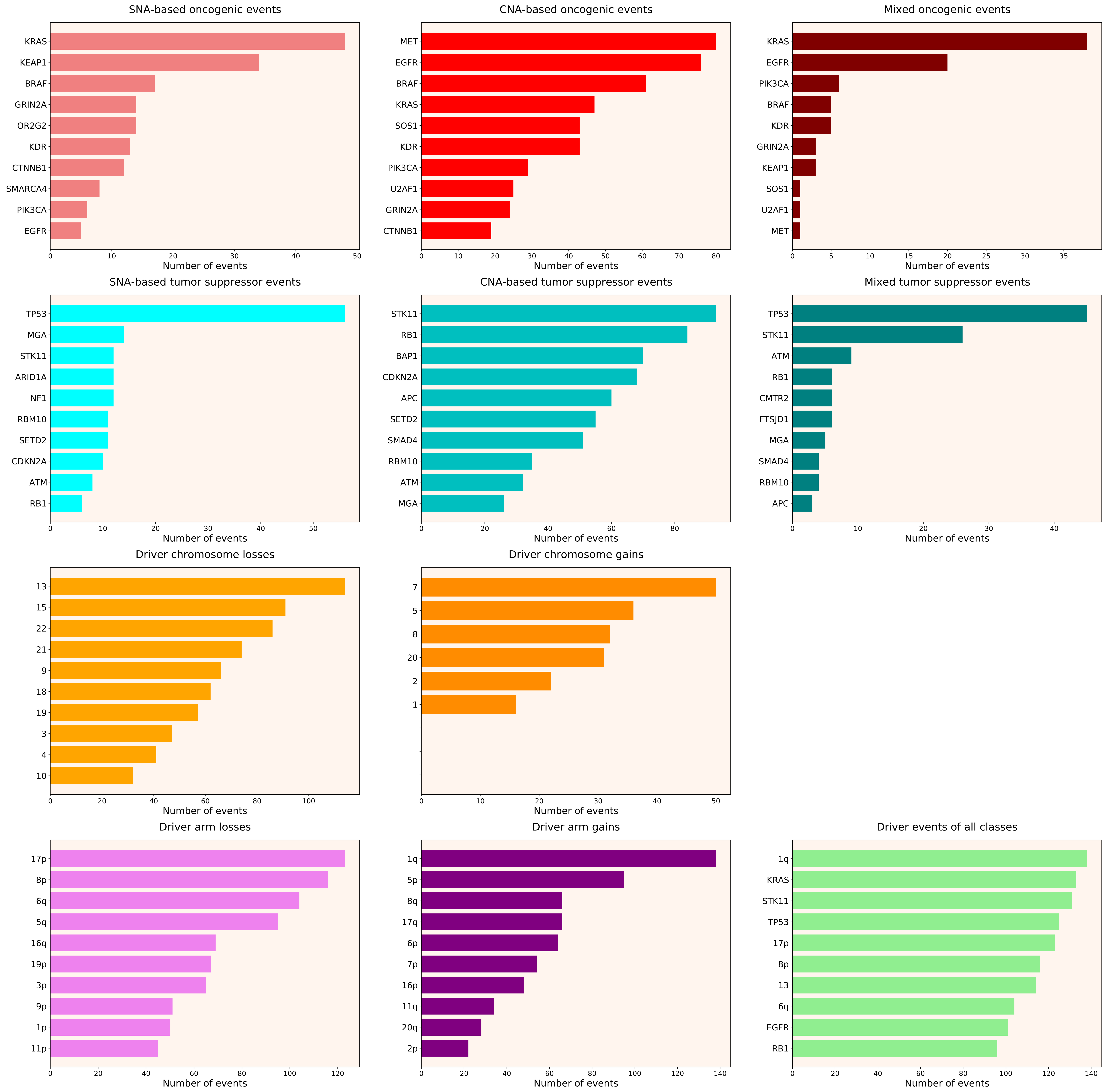

SNA-based oncogenic events
KRAS
KEAP1
BRAF
GRIN2A
OR2G2
KDR
CTNNB1
SMARCA4
PIK3CA
EGFR
Number of events
CNA-based oncogenic events
MET
EGFR
BRAF
KRAS
SOS1
KDR
PIK3CA
U2AF1
GRIN2A
CTNNB1
Number of events
Mixed oncogenic events
KRAS
EGFR
PIK3CA
BRAF
KDR
GRIN2A
KEAP1
SOS1
U2AF1
MET
Number of events
SNA-based tumor suppressor events
TP53
MGA
STK11
ARID1A
NF1
RBM10
SETD2
CDKN2A
ATM
RB1
Number of events
CNA-based tumor suppressor events
STK11
RB1
BAP1
CDKN2A
APC
SETD2
SMAD4
RBM10
ATM
MGA
Number of events
Mixed tumor suppressor events
TP53
STK11
ATM
RB1
CMTR2
FTSJD1
MGA
SMAD4
RBM10
APC
Number of events
Driver chromosome losses
13
15
22
21
9
18
19
3
4
10
Number of events
Driver chromosome gains
7
5
8
20
2
1
Number of events
Driver arm losses
17p
8p
6q
5q
16q
19p
3p
9p
1p
11p
Number of events
Driver arm gains
1q
5p
8q
17q
6p
7p
16p
11q
20q
2p
Number of events
Driver events of all classes
1q
KRAS
STK11
TP53
17p
8p
13
6q
EGFR
RB1
Number of events
